# Methyl *N*-({[2-(2-meth­oxy­acetamido)-4-(phenyl­sulfan­yl)phen­yl]amino}[(meth­oxy­carbonyl)imino]methyl)carbamate

**DOI:** 10.1107/S1600536812002760

**Published:** 2012-02-04

**Authors:** Hoong-Kun Fun, Madhukar Hemamalini, K. Divya, B. Narayana, B. K. Sarojini

**Affiliations:** a X-ray Crystallography Unit, School of Physics, Universiti Sains Malaysia, 11800 USM, Penang, Malaysia; b Department of Studies in Chemistry, Mangalore University, Mangalagangotri 574 199, India; c Department of Chemistry, P. A. College of Engineering, Nadupadavu, Mangalore 574 153, India

## Abstract

In the title compound, C_20_H_22_N_4_O_6_S, the phenyl and benzene rings form a dihedral angle of 58.75 (5)°. Intra­molecular N—H⋯O and N—H⋯N hydrogen bonds generate two *S*(6) and one *S*(7) ring motif, respectively. In the crystal, mol­ecules are linked *via* N—H⋯O, N—H⋯N, C—H⋯S and C—H⋯O hydrogen bonds, forming two-dimensional networks parallel to the *bc* plane.

## Related literature

For the pharmacological properties of febantel, see: Wollweber *et al.* (1978 ▶); Delatour *et al.* (1982 ▶); Su *et al.* (2004 ▶). For a related structure, see: Yıldırım *et al.* (2007 ▶). For hydrogen-bond motifs, see: Bernstein *et al.* (1995 ▶). For bond-length data, see: Allen *et al.* (1987 ▶).
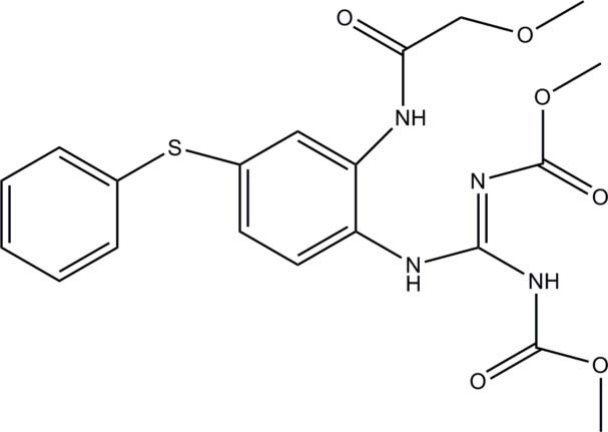


## Experimental

### 

#### Crystal data

C_20_H_22_N_4_O_6_S
*M* *_r_* = 446.48
Monoclinic, 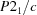

*a* = 10.6975 (7) Å
*b* = 10.6921 (7) Å
*c* = 18.3732 (13) Å
β = 91.068 (1)°
*V* = 2101.1 (2) Å^3^
*Z* = 4
Mo *K*α radiation
μ = 0.20 mm^−1^
*T* = 296 K
0.54 × 0.34 × 0.29 mm

#### Data collection

Bruker APEXII DUO CCD area-detector diffractometer
Absorption correction: multi-scan (*SADABS*; Bruker, 2009 ▶) *T*_min_ = 0.900, *T*_max_ = 0.945
22847 measured reflections
6133 independent reflections
5455 reflections with *I* > 2σ(*I*)
*R*_int_ = 0.023

#### Refinement

*R*[*F* ^2^ > 2σ(*F* ^2^)] = 0.033
*wR*(*F* ^2^) = 0.097
*S* = 1.05
6133 reflections
295 parameters
H atoms treated by a mixture of independent and constrained refinement
Δρ_max_ = 0.38 e Å^−3^
Δρ_min_ = −0.35 e Å^−3^

### 

Data collection: *APEX2* (Bruker, 2009 ▶); cell refinement: *SAINT* (Bruker, 2009 ▶); data reduction: *SAINT*; program(s) used to solve structure: *SHELXTL* (Sheldrick, 2008 ▶); program(s) used to refine structure: *SHELXTL*; molecular graphics: *SHELXTL*; software used to prepare material for publication: *SHELXTL* and *PLATON* (Spek, 2009 ▶).

## Supplementary Material

Crystal structure: contains datablock(s) global, I. DOI: 10.1107/S1600536812002760/rz2701sup1.cif

Structure factors: contains datablock(s) I. DOI: 10.1107/S1600536812002760/rz2701Isup2.hkl

Supplementary material file. DOI: 10.1107/S1600536812002760/rz2701Isup3.cml

Additional supplementary materials:  crystallographic information; 3D view; checkCIF report

## Figures and Tables

**Table 1:** Hydrogen-bond geometry (Å, °)

*D*—H⋯*A*	*D*—H	H⋯*A*	*D*⋯*A*	*D*—H⋯*A*
N4—H1N4⋯N3	0.867 (15)	2.104 (15)	2.8159 (12)	138.9 (13)
N1—H1N1⋯O1	0.848 (16)	2.058 (15)	2.7224 (11)	134.7 (13)
N1—H1N1⋯O5^i^	0.848 (16)	2.425 (16)	3.1162 (11)	139.2 (13)
N2—H1N2⋯O3	0.869 (16)	1.879 (17)	2.6002 (12)	139.3 (15)
C9—H9*A*⋯O3^ii^	0.96	2.38	3.2652 (15)	153
C13—H13*A*⋯S1^iii^	0.97	2.80	3.7678 (11)	179
C14—H14*B*⋯O1^iv^	0.96	2.52	3.3796 (14)	149

Symmetry codes: (i) 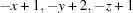; (ii) 
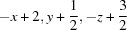
; (iii) 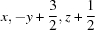; (iv) 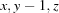.

**Fig. 1.**
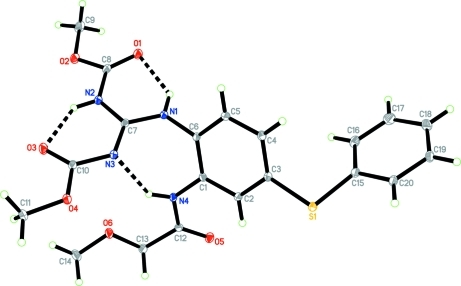
The asymmetric unit of the title compound, showing 30% probability displacement ellipsoids. Intramolecular hydrogen bonds shown by dashed lines.

**Fig. 2.**
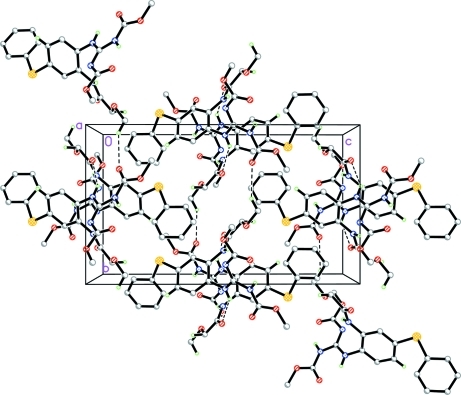
The crystal packing of the title compound viewed along the a axis. H atoms not involved in intermolecular hydrogen bonds (dashed lines) are omitted.

## supplementary crystallographic information

### Comment

Febantel, *N*-{2-[2,3-bis-(methoxycarbonyl)-guanido]-5-(phenylthio)-phenyl}-2-methoxy acetamide, is used as an anthelmintic against gastrointestinal parasites in animals (Wollweber *et al.*, 1978; Su *et al.*, 2004). It is a pro-drug, which get converted into an active compound soon after administration (Delatour *et al.*, 1982). The metabolic pathway of febantel is converted directly to either fenbendazole or oxfendazole, which is achieved via febantel sulfoxide as an intermediate.

The molecular structure of the title compound is shown in Fig. 1. Each of the two intramolecular N—H···O hydrogen bonds generates an *S*(6) ring motif and another intramolecular N—H···N hydrogen bond generates an *S*(7) ring motif (Bernstein *et al.*, 1995). The dihedral angle between the two benzene rings (C1–C6:C15–C20) is 58.75 (5)°. The bond lengths (Allen *et al.*, 1987) and angles are within normal ranges and are comparable to a related structure (Yıldırım *et al.*, 2007).

In the crystal structure (Fig. 2), molecules are linked *via* N—H···O, N—H···N, C—H···S and C—H···O (Table 1) hydrogen bonds, forming two-dimensional networks parallel to the *bc* plane.

### Experimental

A febantel sample was obtained from CAD Pharma Ltd, Bangalore. Crystals of the title compound were obtained from ethanol by slow evaporation method (m.p. 392–395 K).

### Refinement

Atoms H1N1, H1N2 and H1N4 were located from a difference Fourier map and refined freely [N—H = 0.848 (16)–0.868 (17) Å]. The remaining H atoms were positioned geometrically [C—H = 0.93–0.97 Å] and were refined using a riding model, with *U*_iso_(H) = 1.2 or 1.5*U*_eq_(C). A rotating group model was applied to the methyl groups. One outliner, (011), was omitted in the final refinement.

### Crystal data

**Table:**

C_20_H_22_N_4_O_6_S	*F*(000) = 936
*M**_r_* = 446.48	*D*_x_ = 1.411 Mg m^−^^3^
Monoclinic, *P*2_1_/*c*	Mo *K*α radiation, λ = 0.71073 Å
Hall symbol: -P 2ybc	Cell parameters from 9985 reflections
*a* = 10.6975 (7) Å	θ = 2.7–30.1°
*b* = 10.6921 (7) Å	µ = 0.20 mm^−^^1^
*c* = 18.3732 (13) Å	*T* = 296 K
β = 91.068 (1)°	Block, colourless
*V* = 2101.1 (2) Å^3^	0.54 × 0.34 × 0.29 mm
*Z* = 4	

### Data collection

**Table:**

Bruker APEXII DUO CCD area-detector diffractometer	6133 independent reflections
Radiation source: fine-focus sealed tube	5455 reflections with *I* > 2σ(*I*)
Graphite monochromator	*R*_int_ = 0.023
φ and ω scans	θ_max_ = 30.1°, θ_min_ = 1.9°
Absorption correction: multi-scan (*SADABS*; Bruker, 2009)	*h* = −15→15
*T*_min_ = 0.900, *T*_max_ = 0.945	*k* = −14→15
22847 measured reflections	*l* = −25→25

### Refinement

**Table:**

Refinement on *F*^2^	Primary atom site location: structure-invariant direct methods
Least-squares matrix: full	Secondary atom site location: difference Fourier map
*R*[*F*^2^ > 2σ(*F*^2^)] = 0.033	Hydrogen site location: inferred from neighbouring sites
*wR*(*F*^2^) = 0.097	H atoms treated by a mixture of independent and constrained refinement
*S* = 1.05	*w* = 1/[σ^2^(*F*_o_^2^) + (0.0543*P*)^2^ + 0.5625*P*] where *P* = (*F*_o_^2^ + 2*F*_c_^2^)/3
6133 reflections	(Δ/σ)_max_ = 0.001
295 parameters	Δρ_max_ = 0.38 e Å^−^^3^
0 restraints	Δρ_min_ = −0.35 e Å^−^^3^

### Special details

**Table:**

Geometry. All s.u.'s (except the s.u. in the dihedral angle between two l.s. planes) are estimated using the full covariance matrix. The cell s.u.'s are taken into account individually in the estimation of s.u.'s in distances, angles and torsion angles; correlations between s.u.'s in cell parameters are only used when they are defined by crystal symmetry. An approximate (isotropic) treatment of cell s.u.'s is used for estimating s.u.'s involving l.s. planes.
Refinement. Refinement of F^2^ against ALL reflections. The weighted R-factor wR and goodness of fit S are based on F^2^, conventional R-factors R are based on F, with F set to zero for negative F^2^. The threshold expression of F^2^ > 2σ(F^2^) is used only for calculating R-factors(gt) etc. and is not relevant to the choice of reflections for refinement. R-factors based on F^2^ are statistically about twice as large as those based on F, and R- factors based on ALL data will be even larger.

### Fractional atomic coordinates and isotropic or equivalent isotropic displacement parameters (Å^2^)

**Table:**

	*x*	*y*	*z*	*U*_iso_*/*U*_eq_	
S1	0.42127 (3)	0.88419 (2)	0.249402 (14)	0.02036 (7)	
O1	0.77450 (7)	1.22767 (7)	0.60941 (4)	0.02052 (16)	
O2	0.91564 (7)	1.16372 (8)	0.69502 (4)	0.02151 (16)	
O3	1.02647 (7)	0.84660 (8)	0.59724 (4)	0.02269 (16)	
O4	0.99201 (7)	0.71932 (7)	0.50204 (4)	0.01985 (15)	
O5	0.42655 (7)	0.70638 (7)	0.50505 (4)	0.01913 (15)	
O6	0.72299 (7)	0.63922 (7)	0.58737 (4)	0.02042 (16)	
N1	0.72953 (7)	1.04818 (8)	0.50714 (4)	0.01405 (15)	
N2	0.88185 (8)	1.04196 (8)	0.59968 (5)	0.01607 (16)	
N3	0.86819 (7)	0.88433 (8)	0.50935 (5)	0.01461 (16)	
N4	0.61754 (8)	0.80419 (8)	0.50169 (5)	0.01422 (16)	
C1	0.59824 (8)	0.88924 (9)	0.44371 (5)	0.01307 (17)	
C2	0.52211 (9)	0.85788 (9)	0.38387 (5)	0.01432 (17)	
H2A	0.4786	0.7826	0.3835	0.017*	
C3	0.51118 (9)	0.93868 (9)	0.32487 (5)	0.01544 (18)	
C4	0.57771 (9)	1.05126 (10)	0.32423 (5)	0.01744 (19)	
H4A	0.5725	1.1040	0.2840	0.021*	
C5	0.65156 (9)	1.08331 (9)	0.38420 (5)	0.01613 (18)	
H5A	0.6949	1.1587	0.3843	0.019*	
C6	0.66180 (8)	1.00409 (9)	0.44448 (5)	0.01342 (17)	
C7	0.82806 (8)	0.99016 (9)	0.53816 (5)	0.01355 (17)	
C8	0.84971 (9)	1.15290 (10)	0.63288 (5)	0.01655 (18)	
C9	0.89359 (11)	1.27827 (12)	0.73505 (7)	0.0272 (2)	
H9A	0.9310	1.2718	0.7828	0.041*	
H9B	0.8052	1.2914	0.7392	0.041*	
H9C	0.9299	1.3474	0.7097	0.041*	
C10	0.96600 (9)	0.82134 (9)	0.54164 (5)	0.01516 (18)	
C11	1.09167 (10)	0.64191 (10)	0.53104 (6)	0.0216 (2)	
H11A	1.0989	0.5678	0.5019	0.032*	
H11B	1.0735	0.6189	0.5802	0.032*	
H11C	1.1689	0.6875	0.5303	0.032*	
C12	0.53768 (9)	0.71360 (9)	0.52285 (5)	0.01480 (17)	
C13	0.59492 (9)	0.61584 (10)	0.57286 (6)	0.0188 (2)	
H13A	0.5503	0.6150	0.6183	0.023*	
H13B	0.5857	0.5340	0.5506	0.023*	
C14	0.77597 (11)	0.54161 (11)	0.63081 (6)	0.0246 (2)	
H14A	0.8636	0.5570	0.6384	0.037*	
H14B	0.7646	0.4630	0.6063	0.037*	
H14C	0.7353	0.5390	0.6769	0.037*	
C15	0.33882 (9)	1.01680 (9)	0.21622 (5)	0.01638 (18)	
C16	0.29868 (10)	1.11434 (11)	0.26040 (6)	0.0221 (2)	
H16A	0.3188	1.1147	0.3099	0.026*	
C17	0.22839 (11)	1.21108 (11)	0.22990 (7)	0.0276 (2)	
H17A	0.2022	1.2765	0.2593	0.033*	
C18	0.19673 (10)	1.21137 (11)	0.15617 (7)	0.0252 (2)	
H18A	0.1502	1.2768	0.1362	0.030*	
C19	0.23499 (9)	1.11348 (10)	0.11272 (6)	0.0201 (2)	
H19A	0.2127	1.1124	0.0636	0.024*	
C20	0.30667 (9)	1.01637 (10)	0.14207 (5)	0.01720 (18)	
H20A	0.3331	0.9514	0.1124	0.021*	
H1N4	0.6939 (14)	0.7947 (14)	0.5174 (8)	0.026 (4)*	
H1N1	0.7094 (14)	1.1187 (15)	0.5245 (8)	0.024 (4)*	
H1N2	0.9432 (15)	0.9972 (16)	0.6171 (9)	0.032 (4)*	

### Atomic displacement parameters (Å^2^)

**Table:**

	*U*^11^	*U*^22^	*U*^33^	*U*^12^	*U*^13^	*U*^23^
S1	0.03029 (14)	0.01510 (12)	0.01531 (13)	−0.00098 (9)	−0.00951 (9)	0.00060 (9)
O1	0.0204 (3)	0.0201 (4)	0.0209 (4)	0.0025 (3)	−0.0047 (3)	−0.0038 (3)
O2	0.0242 (4)	0.0229 (4)	0.0172 (4)	0.0013 (3)	−0.0074 (3)	−0.0051 (3)
O3	0.0247 (4)	0.0224 (4)	0.0206 (4)	0.0052 (3)	−0.0102 (3)	−0.0019 (3)
O4	0.0179 (3)	0.0178 (3)	0.0236 (4)	0.0047 (3)	−0.0061 (3)	−0.0035 (3)
O5	0.0155 (3)	0.0220 (4)	0.0199 (4)	−0.0032 (3)	−0.0014 (3)	0.0031 (3)
O6	0.0190 (3)	0.0193 (4)	0.0227 (4)	−0.0005 (3)	−0.0054 (3)	0.0077 (3)
N1	0.0145 (3)	0.0132 (4)	0.0144 (4)	0.0004 (3)	−0.0033 (3)	−0.0013 (3)
N2	0.0169 (4)	0.0161 (4)	0.0150 (4)	0.0008 (3)	−0.0050 (3)	−0.0008 (3)
N3	0.0136 (3)	0.0148 (4)	0.0153 (4)	0.0004 (3)	−0.0029 (3)	0.0000 (3)
N4	0.0137 (3)	0.0146 (4)	0.0142 (4)	−0.0011 (3)	−0.0032 (3)	0.0035 (3)
C1	0.0130 (4)	0.0134 (4)	0.0127 (4)	0.0007 (3)	−0.0009 (3)	0.0016 (3)
C2	0.0150 (4)	0.0139 (4)	0.0140 (4)	−0.0010 (3)	−0.0017 (3)	0.0003 (3)
C3	0.0174 (4)	0.0157 (4)	0.0131 (4)	0.0002 (3)	−0.0031 (3)	−0.0003 (3)
C4	0.0214 (4)	0.0167 (4)	0.0141 (4)	−0.0013 (3)	−0.0031 (3)	0.0034 (3)
C5	0.0177 (4)	0.0139 (4)	0.0167 (4)	−0.0017 (3)	−0.0020 (3)	0.0015 (3)
C6	0.0127 (4)	0.0140 (4)	0.0134 (4)	0.0008 (3)	−0.0020 (3)	−0.0004 (3)
C7	0.0131 (4)	0.0145 (4)	0.0130 (4)	−0.0024 (3)	−0.0011 (3)	0.0018 (3)
C8	0.0159 (4)	0.0187 (4)	0.0150 (4)	−0.0031 (3)	−0.0018 (3)	−0.0013 (4)
C9	0.0285 (5)	0.0294 (6)	0.0234 (5)	0.0021 (4)	−0.0071 (4)	−0.0124 (5)
C10	0.0141 (4)	0.0151 (4)	0.0162 (4)	−0.0011 (3)	−0.0011 (3)	0.0015 (3)
C11	0.0178 (4)	0.0182 (5)	0.0287 (6)	0.0046 (4)	−0.0034 (4)	0.0016 (4)
C12	0.0178 (4)	0.0139 (4)	0.0127 (4)	−0.0013 (3)	−0.0003 (3)	0.0005 (3)
C13	0.0197 (4)	0.0176 (5)	0.0189 (5)	−0.0037 (3)	−0.0035 (3)	0.0052 (4)
C14	0.0290 (5)	0.0211 (5)	0.0236 (5)	0.0070 (4)	−0.0038 (4)	0.0050 (4)
C15	0.0159 (4)	0.0175 (4)	0.0156 (4)	−0.0018 (3)	−0.0024 (3)	0.0016 (3)
C16	0.0223 (5)	0.0263 (5)	0.0176 (5)	−0.0002 (4)	−0.0012 (4)	−0.0036 (4)
C17	0.0246 (5)	0.0254 (5)	0.0328 (6)	0.0050 (4)	−0.0005 (4)	−0.0077 (5)
C18	0.0192 (5)	0.0223 (5)	0.0338 (6)	0.0023 (4)	−0.0049 (4)	0.0036 (4)
C19	0.0171 (4)	0.0236 (5)	0.0195 (5)	−0.0022 (4)	−0.0049 (3)	0.0055 (4)
C20	0.0166 (4)	0.0194 (5)	0.0156 (4)	−0.0014 (3)	−0.0024 (3)	0.0004 (4)

### Geometric parameters (Å, º)

**Table:**

S1—C3	1.7712 (10)	C4—C5	1.3868 (14)
S1—C15	1.7720 (10)	C4—H4A	0.9300
O1—C8	1.2081 (13)	C5—C6	1.3971 (13)
O2—C8	1.3357 (12)	C5—H5A	0.9300
O2—C9	1.4502 (14)	C9—H9A	0.9600
O3—C10	1.2289 (12)	C9—H9B	0.9600
O4—C10	1.3433 (12)	C9—H9C	0.9600
O4—C11	1.4438 (12)	C11—H11A	0.9600
O5—C12	1.2296 (12)	C11—H11B	0.9600
O6—C13	1.4132 (12)	C11—H11C	0.9600
O6—C14	1.4249 (13)	C12—C13	1.5136 (14)
N1—C7	1.3410 (12)	C13—H13A	0.9700
N1—C6	1.4288 (12)	C13—H13B	0.9700
N1—H1N1	0.848 (16)	C14—H14A	0.9600
N2—C7	1.3752 (12)	C14—H14B	0.9600
N2—C8	1.3803 (13)	C14—H14C	0.9600
N2—H1N2	0.868 (17)	C15—C16	1.3943 (15)
N3—C7	1.3241 (12)	C15—C20	1.3989 (14)
N3—C10	1.3702 (12)	C16—C17	1.3905 (16)
N4—C12	1.3533 (12)	C16—H16A	0.9300
N4—C1	1.4129 (12)	C17—C18	1.3903 (17)
N4—H1N4	0.867 (15)	C17—H17A	0.9300
C1—C2	1.3968 (13)	C18—C19	1.3830 (17)
C1—C6	1.4036 (13)	C18—H18A	0.9300
C2—C3	1.3894 (13)	C19—C20	1.3933 (14)
C2—H2A	0.9300	C19—H19A	0.9300
C3—C4	1.3986 (14)	C20—H20A	0.9300
			
C3—S1—C15	105.41 (5)	H9B—C9—H9C	109.5
C8—O2—C9	114.73 (9)	O3—C10—O4	121.17 (9)
C10—O4—C11	115.12 (8)	O3—C10—N3	129.63 (9)
C13—O6—C14	110.50 (8)	O4—C10—N3	109.19 (8)
C7—N1—C6	124.93 (8)	O4—C11—H11A	109.5
C7—N1—H1N1	117.1 (10)	O4—C11—H11B	109.5
C6—N1—H1N1	117.9 (10)	H11A—C11—H11B	109.5
C7—N2—C8	127.26 (9)	O4—C11—H11C	109.5
C7—N2—H1N2	112.4 (11)	H11A—C11—H11C	109.5
C8—N2—H1N2	120.3 (11)	H11B—C11—H11C	109.5
C7—N3—C10	119.84 (8)	O5—C12—N4	125.52 (9)
C12—N4—C1	126.38 (8)	O5—C12—C13	119.76 (9)
C12—N4—H1N4	114.6 (10)	N4—C12—C13	114.73 (8)
C1—N4—H1N4	116.7 (10)	O6—C13—C12	111.85 (8)
C2—C1—C6	119.55 (9)	O6—C13—H13A	109.2
C2—C1—N4	120.92 (8)	C12—C13—H13A	109.2
C6—C1—N4	119.44 (8)	O6—C13—H13B	109.2
C3—C2—C1	120.28 (9)	C12—C13—H13B	109.2
C3—C2—H2A	119.9	H13A—C13—H13B	107.9
C1—C2—H2A	119.9	O6—C14—H14A	109.5
C2—C3—C4	120.42 (9)	O6—C14—H14B	109.5
C2—C3—S1	116.29 (7)	H14A—C14—H14B	109.5
C4—C3—S1	123.06 (8)	O6—C14—H14C	109.5
C5—C4—C3	119.24 (9)	H14A—C14—H14C	109.5
C5—C4—H4A	120.4	H14B—C14—H14C	109.5
C3—C4—H4A	120.4	C16—C15—C20	119.78 (9)
C4—C5—C6	121.01 (9)	C16—C15—S1	123.70 (8)
C4—C5—H5A	119.5	C20—C15—S1	116.38 (8)
C6—C5—H5A	119.5	C17—C16—C15	119.46 (10)
C5—C6—C1	119.45 (9)	C17—C16—H16A	120.3
C5—C6—N1	118.02 (8)	C15—C16—H16A	120.3
C1—C6—N1	122.35 (9)	C18—C17—C16	120.96 (11)
N3—C7—N1	118.91 (9)	C18—C17—H17A	119.5
N3—C7—N2	122.61 (9)	C16—C17—H17A	119.5
N1—C7—N2	118.46 (9)	C19—C18—C17	119.43 (10)
O1—C8—O2	125.82 (10)	C19—C18—H18A	120.3
O1—C8—N2	125.50 (9)	C17—C18—H18A	120.3
O2—C8—N2	108.68 (9)	C18—C19—C20	120.48 (10)
O2—C9—H9A	109.5	C18—C19—H19A	119.8
O2—C9—H9B	109.5	C20—C19—H19A	119.8
H9A—C9—H9B	109.5	C19—C20—C15	119.87 (10)
O2—C9—H9C	109.5	C19—C20—H20A	120.1
H9A—C9—H9C	109.5	C15—C20—H20A	120.1
			
C12—N4—C1—C2	−27.56 (14)	C8—N2—C7—N1	−3.23 (15)
C12—N4—C1—C6	155.89 (9)	C9—O2—C8—O1	−1.40 (15)
C6—C1—C2—C3	1.22 (14)	C9—O2—C8—N2	178.50 (9)
N4—C1—C2—C3	−175.32 (9)	C7—N2—C8—O1	−6.22 (17)
C1—C2—C3—C4	1.01 (15)	C7—N2—C8—O2	173.88 (9)
C1—C2—C3—S1	175.56 (7)	C11—O4—C10—O3	1.94 (14)
C15—S1—C3—C2	142.14 (8)	C11—O4—C10—N3	−178.42 (8)
C15—S1—C3—C4	−43.47 (10)	C7—N3—C10—O3	1.18 (16)
C2—C3—C4—C5	−2.11 (15)	C7—N3—C10—O4	−178.42 (8)
S1—C3—C4—C5	−176.27 (8)	C1—N4—C12—O5	−15.45 (16)
C3—C4—C5—C6	0.98 (15)	C1—N4—C12—C13	164.86 (9)
C4—C5—C6—C1	1.23 (14)	C14—O6—C13—C12	−176.40 (9)
C4—C5—C6—N1	−174.02 (9)	O5—C12—C13—O6	−179.96 (9)
C2—C1—C6—C5	−2.32 (14)	N4—C12—C13—O6	−0.25 (13)
N4—C1—C6—C5	174.27 (9)	C3—S1—C15—C16	−31.93 (10)
C2—C1—C6—N1	172.71 (8)	C3—S1—C15—C20	152.41 (8)
N4—C1—C6—N1	−10.69 (13)	C20—C15—C16—C17	−0.84 (16)
C7—N1—C6—C5	−123.35 (10)	S1—C15—C16—C17	−176.36 (9)
C7—N1—C6—C1	61.54 (13)	C15—C16—C17—C18	0.54 (17)
C10—N3—C7—N1	−177.78 (8)	C16—C17—C18—C19	0.49 (18)
C10—N3—C7—N2	0.88 (14)	C17—C18—C19—C20	−1.21 (16)
C6—N1—C7—N3	0.55 (14)	C18—C19—C20—C15	0.90 (15)
C6—N1—C7—N2	−178.17 (8)	C16—C15—C20—C19	0.14 (15)
C8—N2—C7—N3	178.10 (9)	S1—C15—C20—C19	175.98 (8)

### Hydrogen-bond geometry (Å, º)

**Table:**

*D*—H···*A*	*D*—H	H···*A*	*D*···*A*	*D*—H···*A*
N4—H1*N*4···N3	0.867 (15)	2.104 (15)	2.8159 (12)	138.9 (13)
N1—H1*N*1···O1	0.848 (16)	2.058 (15)	2.7224 (11)	134.7 (13)
N1—H1*N*1···O5^i^	0.848 (16)	2.425 (16)	3.1162 (11)	139.2 (13)
N2—H1*N*2···O3	0.869 (16)	1.879 (17)	2.6002 (12)	139.3 (15)
C9—H9*A*···O3^ii^	0.96	2.38	3.2652 (15)	153
C13—H13*A*···S1^iii^	0.97	2.80	3.7678 (11)	179
C14—H14*B*···O1^iv^	0.96	2.52	3.3796 (14)	149

Symmetry codes: (i) −*x*+1, −*y*+2, −*z*+1; (ii) −*x*+2, *y*+1/2, −*z*+3/2; (iii) *x*, −*y*+3/2, *z*+1/2; (iv) *x*, *y*−1, *z*.
